# Tuberculosis of the Pubic Symphysis: A Case Report

**DOI:** 10.7759/cureus.44205

**Published:** 2023-08-27

**Authors:** Mohammed Barrached, Achraf Tebbaa El Hassali, Adnane Lachkar, Najib Abdeljaouad, Hicham Yacoubi

**Affiliations:** 1 Orthopedics and Traumatology, Mohammed VI University Hospital, Oujda, MAR

**Keywords:** anti-tuberculosis therapy, tuberculosis, infection, pubic, adult

## Abstract

Tuberculosis of the pubic symphysis is rare; the diagnosis is often difficult but guided by CT and confirmed by histopathological examination. We report the case of a 25-year-old female with no particular past medical history, presenting with pain at the level of the pubic symphysis. Clinical examination showed a small renitent mass and pain on palpation without inflammatory signs. Radiological investigation showed demineralization, lysis, and diastasis of the pubic symphysis with a cystic image in favor of tuberculosis. A biopsy followed by resection was performed, and histopathology confirmed the diagnosis. The patient received medical care for nine months using the 2RHZE/7RH protocol (rifampicin, isoniazid, pyrazinamide, ethambutol), with good results at follow-up. Bone lesions of the pubic symphysis may exceptionally reveal tuberculosis, and the positive diagnosis is based on a histopathological test.

## Introduction

Tuberculosis of the pubic symphysis is a condition characterized by the presence of tuberculosis infection in the pubic symphysis, which is the joint located at the front of the pelvis where the two pubic bones meet. Tuberculosis is a worldwide public health crisis. The osteoarticular form is uncommon: it represents 2-5 % of all tuberculosis cases [1,2] and 11-15% of extrapulmonary tuberculosis. The main localization remains vertebral tuberculosis, which accounts for half of the cases [3,4]. Pubic localization is particularly rare. Here, we report a case of tuberculosis of the pubic symphysis.

## Case presentation

**Figure 1:**
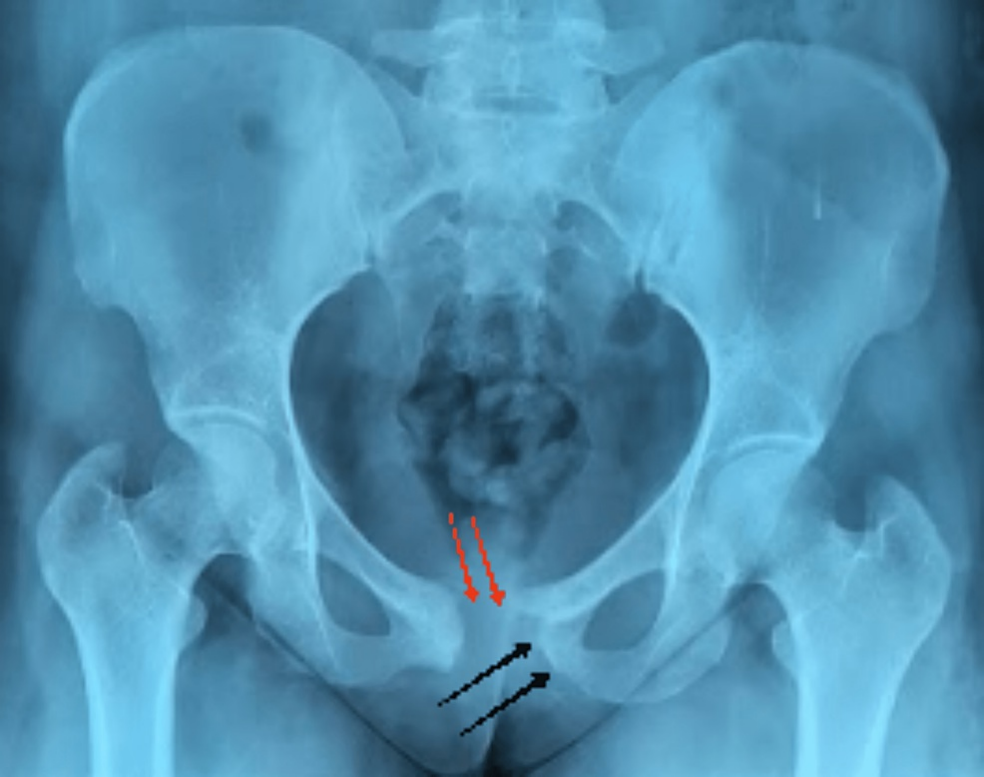
Pelvic X-ray showing a pubic symphysis dislocation (red arrow) with bone lysis of the pubic symphysis margins (black arrows)

**Figure 2:**
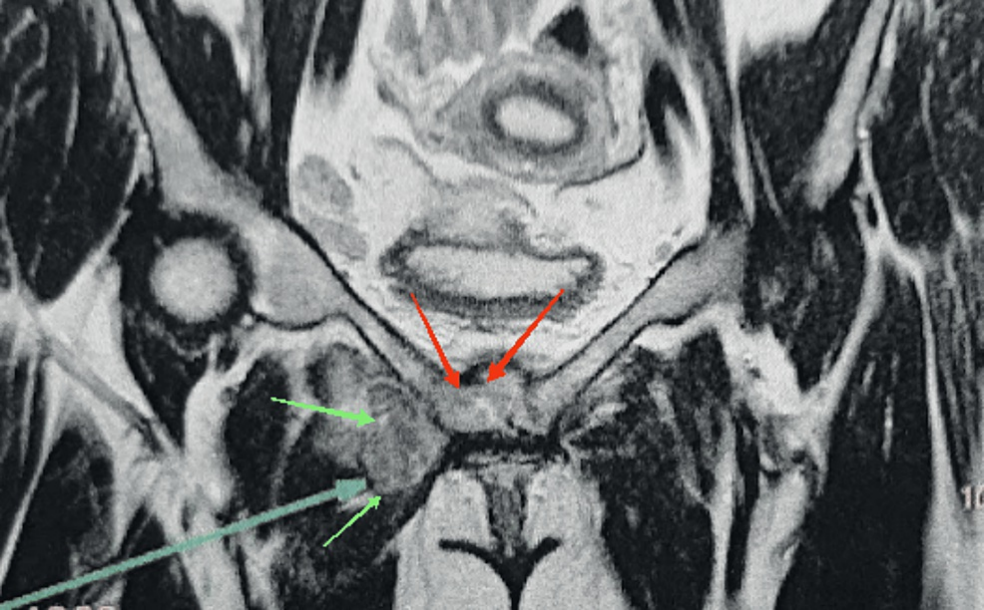
Pelvic MRI showing lysis of the horizontal branch of the pubis (red arrow) and a cystic image opposite (green arrows)

A 25-year-old female patient from eastern Morocco with no past medical history presented with pain localized to the pubic symphysis. The patient reported night sweats without weight loss. Clinical examination revealed a small renitent mass and pain on palpation which is accentuated during the movements of the lower limbs and when walking, without inflammatory signs. No inguinal lymphadenopathy was present. Pelvic X-rays showed lysis of the pubic symphysis margins and diastasis of the pubic symphysis (Figure 1), supplemented by a pelvic MRI that showed lysis and cystic changes to the horizontal branch of the pubis (Figure 2).

**Figure 3:**
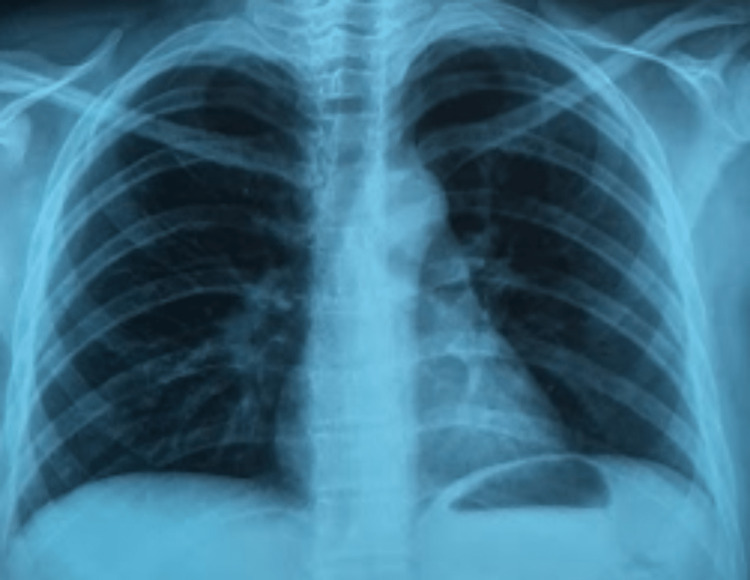
Chest X-ray showing no anomalies

Laboratory tests revealed an inflammatory syndrome with a CRP level of 60 mg/L and hyperleukocytosis of 12000 cells/mm3. Intradermal reaction and acid-fast bacillus smear in sputum were negative, and a chest X-ray showed no abnormalities (Figure 3).

**Figure 4:**
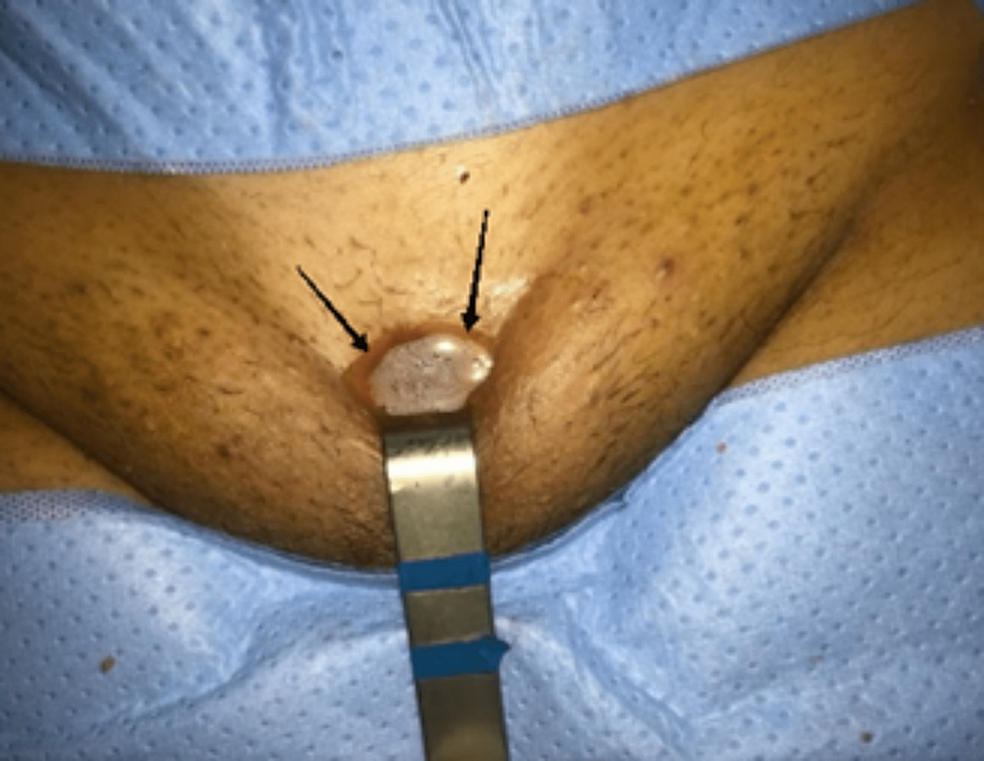
Intraoperative image of the pubic biopsy, surgical approach (black arrows)

The diagnosis of osteoarticular tuberculosis was suspected and was confirmed by histopathological examination of a biopsy of the pubic symphysis (Figure 4).

**Figure 5:**
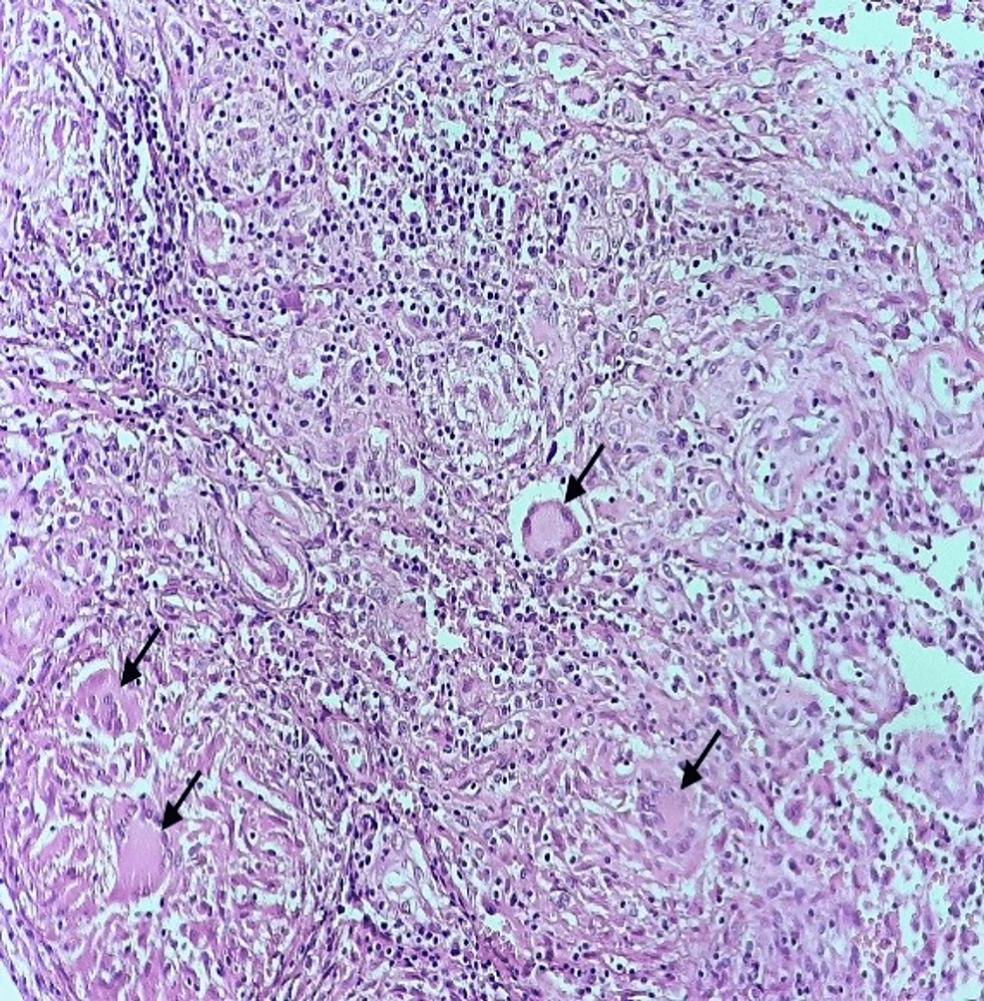
Granulomatous inflammation (black arrows)

**Figure 6:**
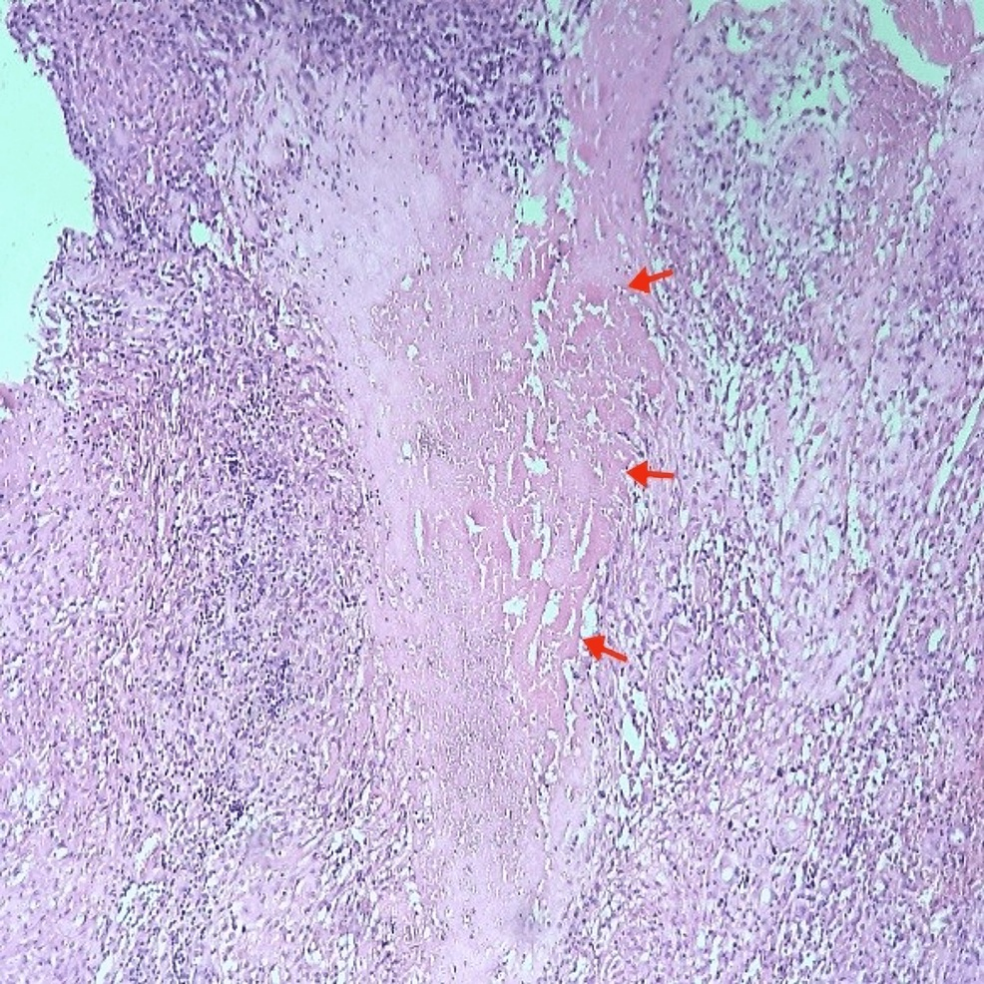
Caseous necrosis (red arrows)

Histological examination of the lesion showed a granulomatous inflammation composed of epithelioid histiocytes and multinucleated giant cells known as Langhans’ cells with surrounding lymphocytes (Figure 5). Characteristic caseating necrosis was also present (Figure 6). Despite negative Ziehl-Neelsen staining for mycobacteria, clinical and histopathological features were very suggestive of symphysis pubis tuberculosis.

The patient received medical treatment for nine months using the 2RHZE/7RH protocol (rifampicin, isoniazid, pyrazinamide, ethambutol). No local or systemic recurrence was noted after 24 months of follow-up.

## Discussion

Osteoarticular tuberculosis represents less than 5% of all tuberculosis cases. Pubic localization is particularly rare, and it affects mostly children and young adults [1,2]. Predisposing factors are poor living conditions, diabetes, advancing age, multiple pregnancies, and immune deficiencies (including human immunodeficiency) [5].

The onset is often insidious and the evolution is slow, ranging from three months to four years [6]. The symptomatology is generally limited to progressive pubic pain, which is accentuated during the movements of the lower limbs and when walking [2] as in this patient. Biological examinations have little diagnostic value. The erythrocyte sedimentation rate is often increased, and we noted in our patient an inflammatory syndrome with a high CRP level. The earliest radiological lesions are diastasis of the pubic symphysis with subchondral bone lysis, followed by irregular lysis of the edges, which can result in significant destruction with a tendency to osteomyelitis [7]. The computed tomography scan is a good examination of tuberculous osteitis, allowing a fine analysis of bone changes and a study of the soft tissues. MRI is particularly useful for the early detection of edema and inflammation of bone and muscle, intra-articular effusion, and abscess.

A definitive diagnosis is based on the identification of the germ: the bone biopsy is the only simple and reliable means of diagnosis. However, in many developing countries where tuberculosis is very common, the diagnosis may be suspected through clinical methods and radiography, which justifies tuberculosis treatment [8]. According to the latest consensus of the World Health Organization, antibiotic therapy is based on the combination of several antituberculosis drugs, the most commonly used of which are rifampicin, isoniazid, pyrazinamide, and ethambutol. Many authors recommend a minimum duration of nine months for their use [9]. Surgery is necessary to drain cold abscesses. The evolution toward healing is manifested by the regression of the abscesses and scarring sclerosis which fills the bone gaps [10].

## Conclusions

Tuberculosis of the pubic symphysis is rare. It is important to diagnose and treat it before the destructive stage. It manifests as chronic pubalgia and is often complicated by soft tissue abscesses.

Bone lesions of the pubic symphysis may exceptionally reveal tuberculosis. A positive diagnosis is based on a histological test. The majority of patients can be treated with anti-tuberculosis drugs, with or without surgery. In general, the evolution is favorable if treatment is well administered.

Tuberculosis remains a global public health problem requiring an obligatory declaration of the disease.
